# Chromosomal 16S Ribosomal RNA Methyltransferase RmtE1 in *Escherichia coli* Sequence Type 448

**DOI:** 10.3201/eid2305.162000

**Published:** 2017-05

**Authors:** Bin Li, Marissa P. Pacey, Yohei Doi

**Affiliations:** Fujian Medical University Union Hospital, Fuzhou, China (B. Li); University of Pittsburgh School of Medicine, Pittsburgh, Pennsylvania, USA (B. Li, M.P. Pacey, Y. Doi);

**Keywords:** RmtE, CMY-2, aminoglycoside resistance, Escherichia coli, bacteria, antimicrobial resistance

## Abstract

We identified *rmtE1*, an uncommon 16S ribosomal methyltransferase gene, in an aminoglycoside- and cephalosporin-resistant *Escherichia coli* sequence type 448 clinical strain co-harboring *bla*_CMY-2_. Long-read sequencing revealed insertion of a 101,257-bp fragment carrying both resistance genes to the chromosome. Our findings underscore *E. coli* sequence type 448 as a potential high-risk multidrug-resistant clone.

RmtE (RmtE1 and its variant RmtE2) is an uncommon plasmid-mediated 16S rRNA methyltransferase (16S RMTase) found in gram-negative bacteria; only 4 strains have been reported to produce RmtE, all *Escherichia coli*, including 1 from the University of Pittsburgh Medical Center (Pittsburgh, PA, USA) (*1*–*3*). We report the genetic context of *rmtE* (*rmtE1*) in another *E. coli* clinical strain identified at this hospital.

*E. coli* YDC774 was identified in 2016 in the urine of a local elderly man with a history of bladder cancer for which he had undergone transurethral resection of the bladder and completed chemotherapy. He had *E. coli* urinary tract infection treated with ciprofloxacin 3 months earlier; further details were unavailable. *E. coli* YDC774 was resistant to cefotaxime, levofloxacin, ciprofloxacin, and trimethoprim/sulfamethoxazole and susceptible to ceftazidime, cefepime, piperacillin/tazobactam, imipenem, meropenem, minocycline, and colistin. The strain was highly resistant to amikacin (MIC >32 μg/mL), gentamycin (MIC >16 μg/mL), and tobramycin (MIC >8 μg/mL). Because the positive culture was believed to represent asymptomatic bacteriuria, the patient was not treated with antimicrobial drugs.

We aimed to elucidate the genetic context of *rmtE* in *E. coli* YDC774. Although *rmtE* has been identified exclusively on plasmids, neither broth conjugation with *E. coli* J53 nor transformation of *E. coli* TOP10 with purified plasmids mobilized *rmtE*, leading us to speculate the gene might be located on the chromosome. We therefore sequenced the YDC774 genome with PacBio RS II sequencing instrument (Pacific Biosciences, Menlo Park, CA, USA) as described (*4*). Sequencing with a single SMRT cell yielded 64,878 reads averaging 10,991 bp. De novo assembly generated 8 contigs; the largest was ≈4.3 Mbp, which had ≈122× coverage and was consistent with a large portion of the *E. coli* chromosome.

*E. coli* YDC774 belonged to sequence type (ST) 448 by in silico multilocus sequence typing. *E. coli* ST448 has been reported in recent years among extended-spectrum β-lactamase– and New Delhi–type metallo-β-lactamase–producing strains (*5*,*6*). The chromosomal contig contained *rmtE* (*rmtE1* allele), *bla*_CMY-2_, *aac(**3**)-VIa*, *aadA*, *strA/B*, *floR*, *sul1*, *sul2*, *tet*(A), and *dfrA7* as determined by ResFinder (https://cge.cbs.dtu.dk/services/ResFinder/). We identified no resistance genes on the other contigs, including those representing a 96-kb IncY plasmid resembling p12579_1 (GenBank accession no. CP003110.1) in enteropathogenic *E. coli* strain RM12579 (99% identity over 83% coverage). Several other 16S RMTase genes, such as *rmtB*, *rmtC*, and *rmtF*, have been found on the chromosome of gram-negative bacteria (*7*,*8*).

The region surrounding *rmtE1* was annotated with Rapid Annotations by using Subsystem Technology server (http://rast.nmpdr.org) and curated manually by using blastn and blastp (http://blast.ncbi.nlm.nih.gov/blast) to elucidate the context of its chromosomal integration. Using *E. coli* ATCC 25922 as the reference genome, we determined that a 101,257-bp sequence was inserted in an intergenic region between the 4′-phosphopantetheinyl transferase gene and the NAD(P)H nitroreductase gene on the *E. coli* chromosome.

This inserted sequence can be divided into 2 regions. The first comprises several inserted sequences, such as IS*186*, IS*CR1*, and 1 antimicrobial resistance gene, *aadA*. Downstream of this first region, the inserted fragment is similar to that in pYDC637, an IncA/C plasmid carrying *rmtE1* also found at the University of Pittsburgh Medical Center in 2012 (Technical Appendix Figure) (*2*). However, the second region comprises 3 small fragments. The first contains *aadA1-bx*, 4 mobile elements, and several other genes and is in reverse orientation from that of pYDC637. The second small fragment harboring *bla*_CMY-2_ is identical to that found in the core region in pYDC637 (Technical Appendix Figure) and also is in reverse orientation from the corresponding region of pYDC637. The third small fragment harboring *rmtE* is located in the acquired region of pYDC637. This finding suggests that, on mobilization into the chromosome, gene rearrangements occurred among these fragments. The region between 2 hypothetical proteins appears to have been deleted at or after integration, which includes genes involved in plasmid replication and conjugative transfer (Technical Appendix Figure).

*rmtE1* is bound by an IS*CR20*-like element and an IS*1294*-like insertion sequence. This immediate unit is identical to that found in pYDC637. IS*CR20* and IS*1294* belong to IS*91* family, which is considered related to some antimicrobial drug resistance genes, including 16S RMTase genes, which appears to have been the case in the mobilization of *rmtE1* as well. We could not identify direct repeats upstream and downstream of the unit that would define the exact boundary of this unit. In comparing the genetic context of *rmtE1* and *rmtE2*, IS*CR20*-like transposase is located upstream of *rmtE1* and *rmtE2* (GenBank accession nos. KT428293 and NZ_LRIX01000127). However, the transposase genes located downstream of the 2 16S RMTase genes are distinct. The genetic environment of *rmtE2* is identical between the 2 plasmids from China (GenBank accession no. KT428293) and Canada (GenBank accession no. NZ_LRIX01000127).

In summary, we identified chromosomal integration of *rmtE1*, an unusual 16S RMTase, and *bla*_CMY-2_, a commonly observed acquired AmpC β-lactamase, in an *E. coli* ST448 clinical strain, an event that generated stable co-resistance to aminoglycosides and oxyiminocephalosporins. We found no evidence of further spread of this strain in the hospital. Nonetheless, the findings underscore *E. coli* ST448 as a potential high-risk multidrug-resistant *E. coli* clone.

Technical Appendix Comparative analysis of the rmtE1-carrying region of the chromosome of *Escherichia coli* YDC774 and pYDC637 from *E. coli* YDC637.
